# Sex modifies the effect of genetic risk scores for polycystic ovary syndrome on metabolic phenotypes

**DOI:** 10.1371/journal.pgen.1010764

**Published:** 2023-05-31

**Authors:** Ky’Era V. Actkins, Genevieve Jean-Pierre, Melinda C. Aldrich, Digna R. Velez Edwards, Lea K. Davis

**Affiliations:** 1 Epidemiology Branch, National Institute of Environmental Health Sciences, Research Triangle Park, North Carolina, United States of America; 2 Vanderbilt Genetics Institute, Vanderbilt University Medical Center, Nashville, Tennessee, United States of America; 3 Division of Genetic Medicine, Department of Medicine, Vanderbilt University Medical Center, Nashville, Tennessee, United States of America; 4 Department of Thoracic Surgery, Vanderbilt University Medical Center, Nashville, Tennessee, United States of America; 5 Department of Biomedical Informatics, Vanderbilt University Medical Center, Nashville, Tennessee, United States of America; 6 Vanderbilt Epidemiology Center, Institute of Medicine and Public Health, Vanderbilt University Medical Center, Nashville, Tennessee, United States of America; 7 Division of Quantitative Sciences, Department of Obstetrics and Gynecology, Vanderbilt University Medical Center, Nashville, Tennessee, United States of America; Northwestern University, UNITED STATES

## Abstract

Females with polycystic ovary syndrome (PCOS), the most common endocrine disorder in women, have an increased risk of developing cardiometabolic disorders such as insulin resistance, obesity, and type 2 diabetes (T2D). While only diagnosable in females, males with a family history of PCOS can also exhibit a poor cardiometabolic profile. Therefore, we aimed to elucidate the role of sex in the cardiometabolic comorbidities observed in PCOS by conducting bidirectional genetic risk score analyses in both sexes. We first conducted a phenome-wide association study (PheWAS) using PCOS polygenic risk scores (PCOS_PRS_) to identify potential pleiotropic effects of PCOS_PRS_ across 1,380 medical conditions recorded in the Vanderbilt University Medical Center electronic health record (EHR) database, in females and males. After adjusting for age and genetic ancestry, we found that European (EUR)-ancestry males with higher PCOS_PRS_ were significantly more likely to develop hypertensive diseases than females at the same level of genetic risk. We performed the same analysis in an African (AFR)-ancestry population, but observed no significant associations, likely due to poor trans-ancestry performance of the PRS. Based on observed significant associations in the EUR-ancestry population, we then tested whether the PRS for comorbid conditions (e.g., T2D, body mass index (BMI), hypertension, etc.) also increased the odds of a PCOS diagnosis. Only BMI_PRS_ and T2D_PRS_ were significantly associated with a PCOS diagnosis in EUR-ancestry females. We then further adjusted the T2D_PRS_ for measured BMI and BMI_residual_ (regressed on the BMI_PRS_ and enriched for the environmental contribution to BMI). Results demonstrated that genetically regulated BMI primarily accounted for the relationship between T2D_PRS_ and PCOS. Overall, our findings show that the genetic architecture of PCOS has distinct sex differences in associations with genetically correlated cardiometabolic traits. It is possible that the cardiometabolic comorbidities observed in PCOS are primarily explained by their shared genetic risk factors, which can be further influenced by biological variables including sex and BMI.

## Author summary

Polycystic ovary syndrome (PCOS) is a heterogeneous endocrine disorder in reproductive age females characterized by reproductive and metabolic dysregulation. Despite only being diagnosable in females, males with a family history of PCOS can also present with unfavorable cardiometabolic outcomes. Here, we show that the genetic risk of PCOS (PCOS_PRS_) can lead to sex-specific comorbidities, in which males are more likely to develop cardiovascular diseases and females are more likely to develop type 2 diabetes (T2D). Although many of these associations were affected by body mass index (BMI), genetically regulated BMI was specifically revealed as the primary driver between T2D and PCOS. In conclusion, we showed that the link between PCOS and cardiometabolic comorbidities can be explained by mediating genetic factors that can be further modified by biological factors such as sex and BMI.

## Introduction

Polycystic ovary syndrome (PCOS) is a highly heritable endocrine disorder that affects 5%-21% of females of reproductive age who are typically diagnosed by having two or more of the following features under the Rotterdam criteria: polycystic ovaries, oligo- and anovulation, or hyperandrogenism [1–3]. Although Rotterdam is the most common PCOS criteria, other criteria can be used for diagnosis, including the National Institutes of Health criteria, the Androgen Excess and PCOS Criteria, or the 2018 International Evidence Based PCOS guidelines [2]. Each criterion slightly differs in requirements in an effort to cover the range of PCOS symptoms that are exhibited in patients. However, as a result of the differing diagnostic criteria, the heterogenous presentation of symptoms, and the prevalent comorbidities that reside outside of diagnostic requirements, patients often spend years seeking a diagnosis or worse, may be one of the 75% of females estimated with PCOS who are undiagnosed [4,5].

Many clinicians select criteria based on their perception of the most defining PCOS feature [6]. In some cases, as with the Androgen Excess and PCOS Society criteria [7], this will mean hyperandrogenism, a symptom that typically manifests as acne, hirsutism, or alopecia [8]. Androgen excess is also hypothesized to underlie many of the comorbid metabolic dysfunctions experienced by patients such as insulin resistance, obesity, metabolic syndrome, type 2 diabetes (T2D), and cardiometabolic diseases (CMDs). However, as previous studies have shown, the genetic risk factors present in patients with metabolic manifestations of PCOS could differ from others who have primary presentations of reproductive dysfunction [9,10].

PCOS is multifactorial and twin studies estimate heritability at 70% [11–14]. With an underlying polygenic architecture, multiple variants are hypothesized to be involved in the development of PCOS [15,16]. Furthermore, the variation of clinical features can be partially explained by ancestry informative markers, indicating population specific genotypes may be correlated with PCOS [17,18]. Despite the small effect size of individual common variants, aggregation of common risk variants together as a polygenic (or genetic) risk score (PRS) reflects the overall additive genetic liability to PCOS. This marker of disease risk is associated with PCOS diagnosis in multiple ancestries and offers many advantages to parsing out the genetic etiology of PCOS that is entangled with its comorbid presentations [11–13]. Furthermore, there is increasing evidence that a spectrum of clinical PCOS manifestations is also correlated with the PCOS_PRS_ [11].

Therefore, in this study, we aimed to determine whether the PCOS_PRS_ demonstrated pleiotropic associations with other health conditions in a hospital biobank population through a phenome-wide association study (PheWAS). We then tested the PCOS_PRS_ in males and females separately revealing the impact of PCOS-associated inherited genetic variation in males, despite the fact that clinical PCOS is only diagnosed in females. Guided by the results of the PheWAS, we further performed additional analyses to determine whether genetic risk for associated cardiometabolic phenotypes also increases risk of PCOS. Lastly, we characterized the impact of body mass index (BMI) on the genetic relationship between PCOS and cardiometabolic comorbidities.

## Methods

### VUMC EHR-linked biorepository

This study used a retrospective cohort design including individuals who visited Vanderbilt University Medical Center (VUMC) between January 1990 to February 2020. VUMC is a tertiary care hospital in Nashville, Tennessee, with several outpatient clinics throughout Tennessee and the surrounding states offering primary and secondary care. Medical records have been electronically documented at VUMC since 1990, resulting in a clinical research database of over 3 million EHRs referred to as the Synthetic Derivative [19]. EHRs include demographic information, health information documented through International Classification of Disease, Ninth Revision (ICD9) and Tenth Revision (ICD10) codes, procedural codes (CPT), clinical notes, medications, and laboratory values. This information is linked with a DNA biorepository known as BioVU. Use of EHRs and genetic data for this study was approved by the Vanderbilt University Institutional Review Board (IRB #160279).

### Genetic data

BioVU contains 94,474 individuals genotyped on the MEGA^EX^ platform, which was designed with an increased number of variants found in diverse ancestries to improve genotyping coverage in these populations [20]. We applied a standard quality control pipeline which removed SNPs with low genotyping call rate (< 0.98) and individuals who were related (pi-hat > 0.2), had low call rates (< 0.98), sex discrepancies, or excessive heterozygosity (Fhet > 0.2). A principal component (PC) analysis (PCA) was performed on remaining individuals to determine genetic ancestry using FlashPCA2 [21]. BioVU genotyped samples were stratified by ancestral origin based on PCs herein referred to as the European (EUR) or African (AFR) ancestry dataset. Extended details for the quality control of these datasets have been described previously [22].

### Generation of polygenic risk scores (PRS) for PCOS

PCOS_PRS_ were calculated with PRS-CS software using the weighted sums of the risk allele effects as reported in the summary statistics from the Day et al. GWAS of PCOS and applying a Bayesian continuous shrinkage parameter to select SNP features and to model linkage disequilibrium [16,23]. We calculated PCOS_PRS_ for both EUR and AFR BioVU genotyped ancestry samples. We previously demonstrated the PCOS_PRS_ is associated with a PCOS diagnosis defined by our previously published EHR-based algorithm [11]. PCOS_PRS_ were trained on the full PCOS GWAS dataset (N_SNPs-EUR_ = 777,507, N_SNPs-AFR_ = 766,260) and the limited dataset that included only the most significantly associated 10,000 SNPs, due to the restrictions on data sharing when including participants from 23andMe (N_SNPs-EUR_ = 1,210, N_SNPs-AFR_ = 1,192).

### Phenome-wide association study (PheWAS) of PCOS_PRS_

Next, we were interested in identifying the pleiotropic effects of the genetic susceptibility to PCOS on the medical phenome. Therefore, we analyzed the effects of PCOS_PRS_ across 1,380 medical conditions using a PheWAS framework in which multivariable logistic regressions were performed with PCOS_PRS_ as the indicator variable. This analysis was first performed in a sex-combined sample for our EUR and AFR-ancestry datasets. While males cannot be diagnosed with PCOS, they still harbor genetic risk for PCOS and thus, were included. In the sex-combined logistic regression model, covariates included the median age of individuals across their medical record, sex, and the first ten principal components (PCs) estimated from genetic data to control for ancestry. Next, females and males were analyzed separately. In the sex-stratified models, covariates included median age across an individuals’ EHR and the first ten PCs.

### Sex interaction analysis for phenotypes with significant PCOS_PRS_ main effects

For each phenotype with evidence of a significant main effect of PCOS_PRS_ in either sex (defined as false discovery rate [FDR] q < 0.05), we tested for two-way interactions (sex * PCOS_PRS_) to determine whether the phenome-wide PCOS_PRS_ associations were influenced by biological effects of sex. These selected phenotypes included hypertension, essential hypertension, hypertensive heart disease, coronary atherosclerosis, ischemic heart disease, loss of teeth or edentulism, obesity, type 2 diabetes, diabetes mellitus, and overweight, obesity, and other hyperalimentation. For these interaction analyses, sex-combined models included the multiplicative effects of sex*PCOS_PRS_, main effects of sex, PCOS_PRS_, median age of individuals across their medical record, and the top ten PCs.

Additionally, we tested for the modifying effects of sex on BMI for the same significant traits observed in the PheWAS analysis. This model included the multiplicative effects of sex*BMI, main effects of sex, median BMI of individuals across their medical record, PCOS_PRS_, median age of individuals across their medical record, and the top ten PCs.

### PheWAS sensitivity analyses

Several sensitivity analyses were performed to assess the robustness of the significant phenome-wide findings. First, we evaluated which phenotypes were independent of a PCOS diagnosis in females by accounting for PCOS diagnosis [11]. This analysis allowed us to identify true pleiotropic associations and provided insight into which phenotypes were exclusively correlated with genetic risk, even in the absence of PCOS.

BMI is strongly correlated with both PCOS and its comorbidities, and thus, can influence the strength of the results [24]. Therefore, we adjusted for BMI (median measurement across an individual’s EHR) to test whether the significant phenotypes associated with PCOS_PRS_ were independent of obesity-related effects. In addition to adjusting for BMI, the models were adjusted for median age, sex (only in the sex-combined sample), and the top ten PCs.

Finally, given that BMI has strong contributions from both genetic and environmental sources of variance, we specifically accounted for the environmental contribution to BMI. To do this, we calculated the residuals of individuals’ median BMI (i.e., median measurement across the medical record) adjusted for BMI_PRS_ (residuals(medianBMI ~ BMI_PRS_)) and used it as a covariate in a subsequent sensitivity analysis of the previously described PheWAS. This residual BMI variable is herein referred to as BMI_residual_.

### Publicly available summary statistics for cardiometabolic disorders (CMDs)

Genome-wide association study (GWAS) summary statistics were acquired for BMI (as a proxy for obesity), diastolic blood pressure, systolic blood pressure, pulse pressure, T2D, heart failure, and coronary artery disease (i.e., representing each of the significant phenotypic associations observed in the PheWAS models described below). Each GWAS was selected based on public availability, maximal sample size, and maximal sample diversity.

The Genetic Investigation of Anthropometric Traits (GIANT) consortium BMI summary statistics included 339,224 individuals of European and non-European ancestry [25]. Blood pressure traits (diastolic, systolic, and pulse) and type 2 diabetes (T2D) summary statistics were obtained from the Million Veteran Program (MVP), a large biobank consortium effort that houses biobank data from various sites in the Department of Veterans Affairs health system [26]. Blood pressure traits were generated from a trans-ethnic sample of over 750,000 individuals from MVP [27]. T2D summary statistics were generated from a meta-analysis using data from 1.4 million participants in various biobanks and consortia groups [28]. Heart failure summary statistics were collected from 47,309 cases and 930,014 controls of European ancestry across nine studies in the Heart Failure Molecular Epidemiology for Therapeutic Targets (HERMES) consortium [29]. Finally, coronary artery disease (CAD) datasets generated from the Coronary Artery Disease Genome-wide Replication and Meta-analysis plus The Coronary Artery Disease (CARDIoGRAMplusC4D) consortium were used as the genetic measurement for the coronary atherosclerosis phenotype [30]. This meta-analysis assembled 60,801 cases and 123,504 controls of multiple ancestries across forty-eight study sites.

### Genetic correlation between PCOS and CMDs

Linkage Disequilibrium Score Regression (LDSC) was used to calculate genetic correlation, an estimate of genetic similarity, between traits [31]. LDSC only utilizes GWAS summary statistics and is not sensitive to sample overlap, which may be present across the publicly available GWAS datasets used in this study. This method utilizes the effect estimate of each SNP to estimate genetic correlation between traits while accounting for the effects of SNPs in linkage disequilibrium based on the GWAS reference population. The European reference panel was used for all analyses based on the demographic majority of each of the GWAS samples.

### Logistic regression models testing CMD_PRS_ associations with PCOS diagnosis

Publicly available GWAS summary statistics were used to generate PRS for phenome-wide significant phenotypes identified in the PheWAS. The full set of GWAS summary statistics for each trait was used to construct PRS in the EUR and AFR datasets for T2D (N_SNPs-EUR_ = 780,648, N_SNPs-AFR_ = 769,459), BMI (N_SNPs-EUR_ = 687,279, N_SNPs-AFR_ = 677,322), systolic blood pressure (N_SNPs-EUR_ = 784,750, N_SNPs-AFR_ = 773,483), diastolic blood pressure (N_SNPs-EUR_ = 784,746, N_SNPs-AFR_ = 773,479), pulse pressure (N_SNPs-EUR_ = 784,753, N_SNPs-AFR_ = 773,486), CAD (N_SNPs-EUR_ = 776,073, N_SNPs-AFR_ = 761,746), and heart failure (N_SNPs-EUR_ = 778,308, N_SNPs-AFR_ = 767,048).

BioVU genotyped datasets were filtered to females and contained 365 PCOS cases and 6,597 controls in the EUR dataset and 149 PCOS cases and 2,182 controls in the AFR dataset. In brief, cases required PCOS billing codes for polycystic ovaries or irregular menstruation and hirsutism, and no exclusion codes (i.e., coded strict algorithm). Exclusions were comprised of codes that could affect menstruation cycles and mimic PCOS symptoms such as Cushing’s syndrome. For a complete list, see the supplementary material found in Actkins et al. 2020 [11]. Controls excluded individuals who had any inclusion or exclusion codes. The algorithmic positive predictive value was 96%, the sensitivity was 68%, and the specificity was 25% when compared to a gold standard of clinician adjudicated chart review of 200 patients meeting a data floor and filter criteria [11]. Additional details regarding the selection of PCOS cases and controls in the VUMC EHR dataset have been described elsewhere [11].

Each CMD_PRS_ was used as the independent variable in a logistic regression model with algorithmically defined PCOS diagnosis as the dependent variable. All models were adjusted for median age of the individuals’ medical record and the top ten PCs for each ancestry. BMI was included as a covariate in the sensitivity analysis.

Lastly, we adjusted for BMI_residual_, instead of clinically measured BMI, in the logistic regression models. A multiple testing correction of p < 1.16x10^-3^ (0.05/43) was implemented to account for all statistical tests in the PCOS_PRS_ sex interaction analysis, BMI and sex interaction analysis, genetic correlation analysis, and CMD_PRS_ regression analysis for EUR-ancestry females. All statistical analyses were done using R 3.6.0.

## Results

### Sex combined PCOS_PRS_ PheWAS results

**Fig 1:**
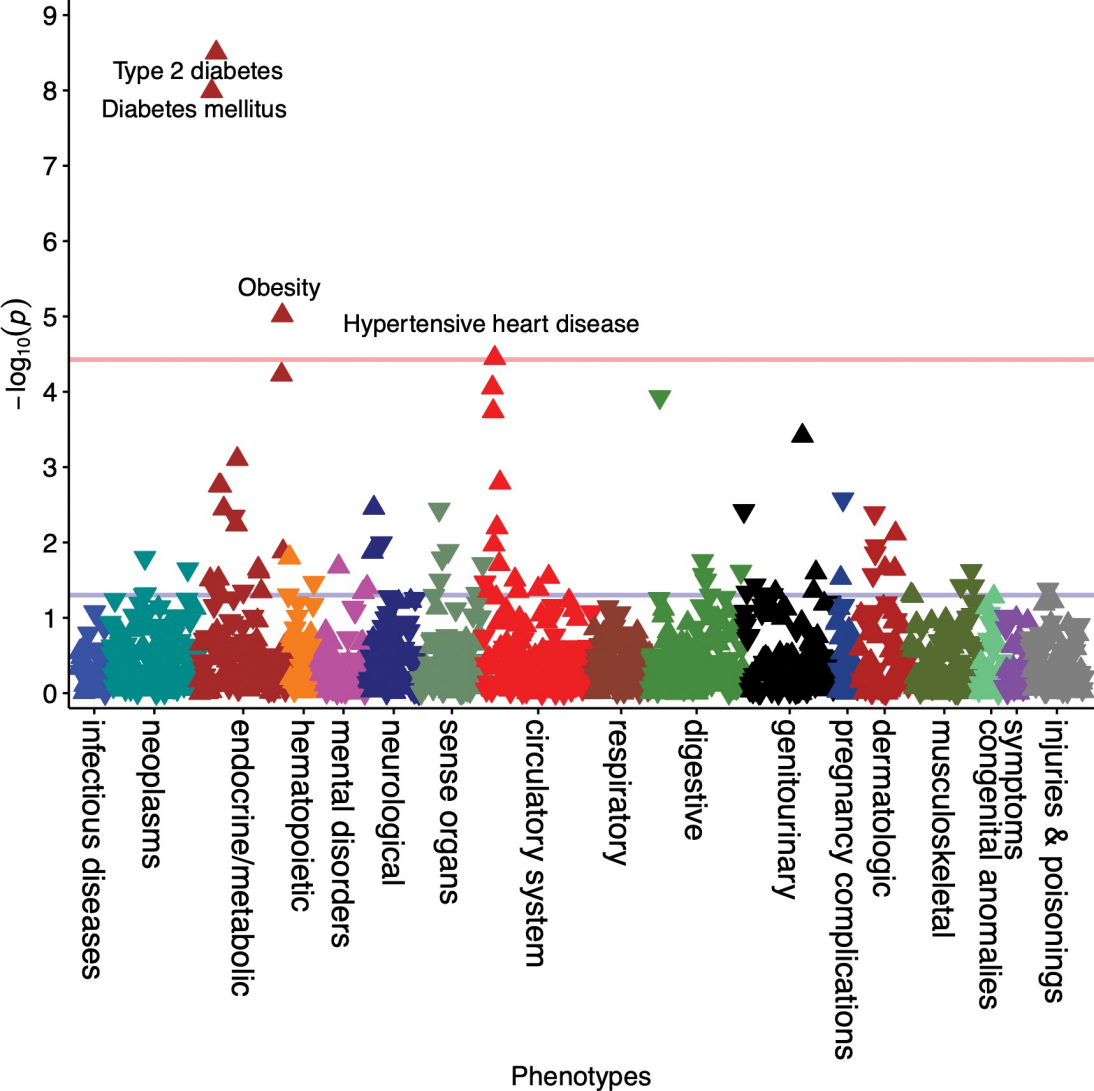
PCOS_PRS_ is Associated with Cardiometabolic Traits. The phenome-wide association study (PheWAS) was performed on the full dataset (females and males) to increase power. Significant results that passed the Bonferroni correction (P = 3.73x10^-5^) are annotated in the Manhattan plot. Arrows pointing upward represent increased risk.

In the EUR-ancestry sex-combined model, four phenotypes were significantly associated with PCOS_PRS_ (**Fig 1**). This included T2D (odds ratio [OR] = 1.08, 95% confidence interval [CI] = 1.06–1.11, p = 3.15x10^-9^), diabetes mellitus (OR = 1.08, 95% CI = 1.05–1.11, p = 1.03x10^-8^), obesity (OR = 1.07, 95% CI = 1.04–1.11, p = 9.68x10^-6^), and hypertensive heart disease (OR = 1.11, 95% CI = 1.06–1.16, p = 3.57x10^-5^). PCOS_PRS_ yielded an OR of 1.21 (95% CI = 1.08–1.36, p = 7.82x10^-4^) with polycystic ovaries, falling just short of phenome-wide significance in the PCOS_PRS_ built using the full GWAS dataset. Although PCOS_PRS_ had a larger effect on polycystic ovaries (OR = 1.21) compared to T2D (OR = 1.08), the p-value was smaller for T2D because it is a more common condition and thus, had a larger case sample size in the PheWAS analysis (N_cases_ = 8,990) compared to polycystic ovaries (N_cases_ = 400). When restricted to the PCOS_PRS_ built using the top 10k SNPs, polycystic ovaries, ovarian dysfunction, and other disorders of stomach and duodenum were significantly associated with PCOS_PRS_ (**S1 Fig**)_._

No associations passed Bonferroni correction in the sex-combined AFR-ancestry analysis when using the full GWAS dataset or the top 10k SNPs, which included a total of 12,383 individuals (**S2 Fig and S1 Supporting Information**). However, top associations from this analysis showed expected PCOS comorbidities including sleep disorders, obesity, and adrenal gland disorders.

### Sex stratified PCOS_PRS_ PheWAS results

**Fig 2:**
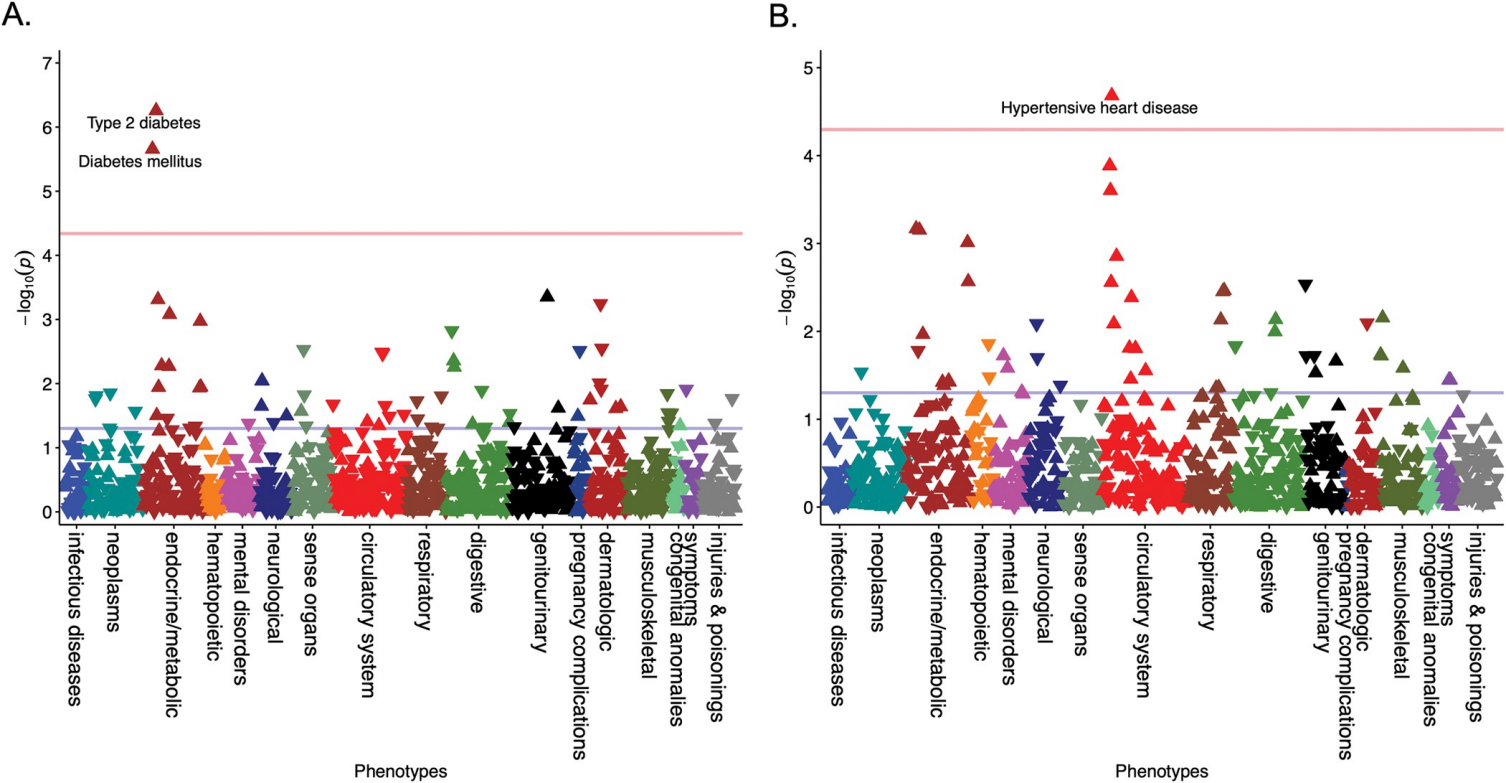
Cardiometabolic Associations Differ by Sex for Genetically High-Risk Individuals. PCOS_PRS_ phenome-wide association study results are displayed in the Manhattan plots for (A) females and (B) males. The red line represents the Bonferroni correction for females (P = 4.57x10^-5^) and males (P = 5.05x10^-5^), respectively. Arrows pointing upward represent increased risk.

In the EUR-ancestry female stratified analysis (N = 37,240), two phenotypes were significantly associated with PCOS_PRS_ (Bonferroni corrected p-value = 4.57x10^-5^) (**Fig 2A**): T2D (OR = 1.10, 95% CI = 1.06–1.14, p = 5.54x10^-7^) and diabetes mellitus (OR = 1.09, 95% CI = 1.05–1.13, p = 2.20x10^-6^). In the EUR-ancestry male stratified analysis (N = 29,663, Bonferroni correction p-value = 5.05x10^-5^; **Fig 2B**), hypertensive heart disease was significant (OR = 1.15, 95% CI = 1.08–1.23, p = 2.07x10^-5^) alongside a cluster of nominally associated cardiovascular phenotypes. This included hypertension (OR = 1.06, 95% CI = 1.03–1.10, p = 1.30x10^-4^), essential hypertension (OR = 1.06, 95% CI = 1.03–1.10, p = 2.49x10^-4^), and coronary atherosclerosis (OR = 1.06, 95% CI = 1.02–1.10, p = 1.40x10^-3^). T2D (OR = 1.07, 95% CI = 1.03–1.11, p = 7.04x10^-4^) and several other endocrine phenotypes were also modestly associated with PCOS_PRS_ in males.

No associations in the sex-stratified AFR-ancestry analysis reached statistical significance after correction for multiple testing (**S1 Supporting Information**).

### Sex interaction analysis of significant PCOS_PRS_ PheWAS associations

**Fig 3:**
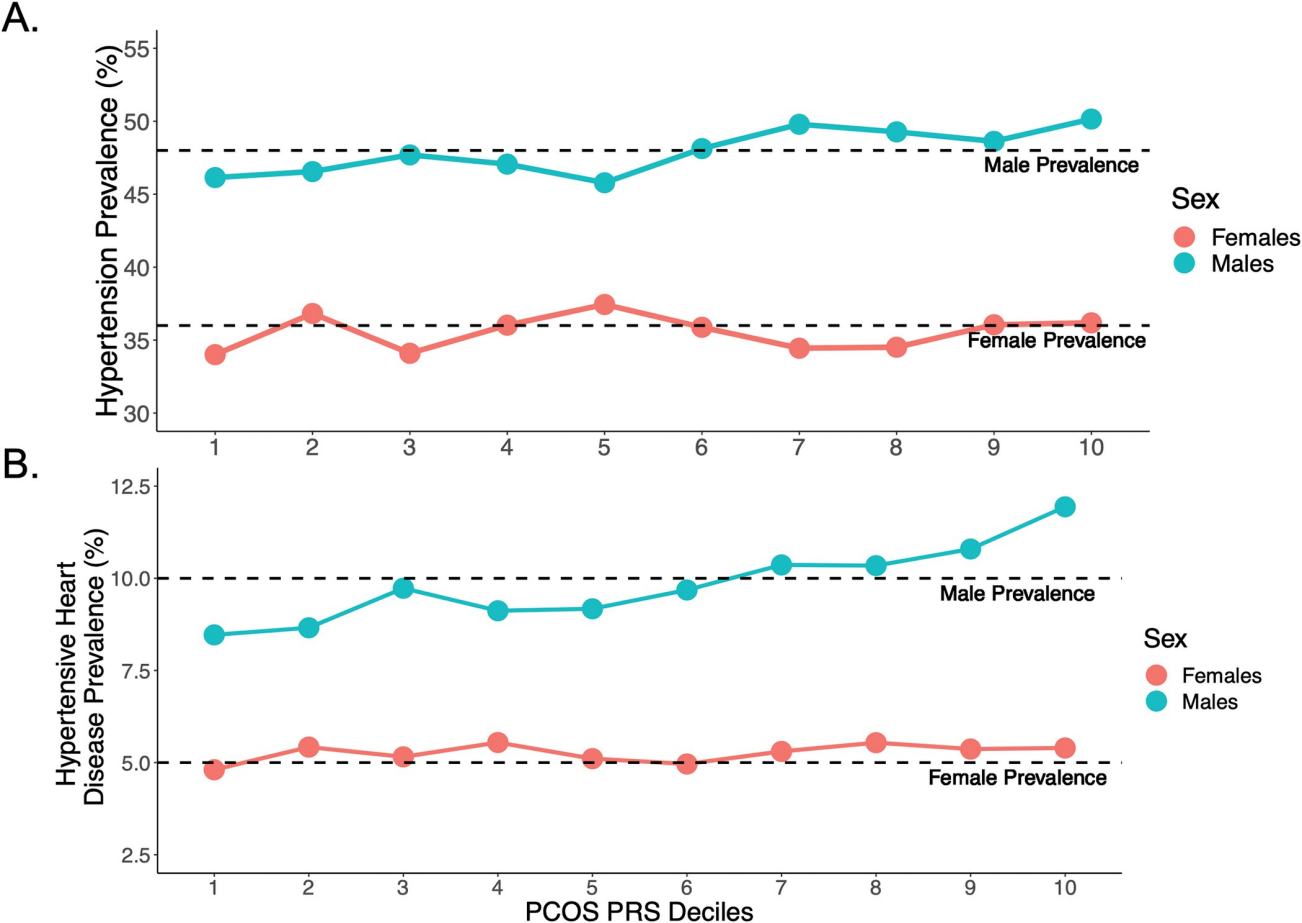
The Prevalence of Hypertensive Traits Differs Between Sexes and Across the PCOS_PRS_ Strata. (A) Hypertension and (B) hypertensive heart disease were significant in the PCOS_PRS_ by sex interaction analysis. The prevalence for hypertension in our dataset was 48% for males and 36% for females. The prevalence for hypertensive heart disease was 10% for males and 5% for females.

The sex interaction analysis demonstrated that males of EUR-ancestry with a high PCOS_PRS_ were more likely to be diagnosed with hypertension (p_interaction_ = 7.24x10^-3^), essential hypertension (p_interaction_ = 7.71x10^-3^), and hypertensive heart disease (p_interaction_ = 1.19x10^-2^) than females with the same PCOS_PRS_ when an FDR threshold of 0.05 was implemented (**Table 1**). These sex differences were also observed when calculating the prevalence for each trait by decile of PCOS_PRS_ (**Fig 3**).

**Table 1:** Hypertension is Significantly Modified by Sex for PCOS_PRS_.

	Males				Females				
Phenotype	OR	LCI	UCI	p	OR	LCI	UCI	p	Interaction P Value
Hypertension	1.06	1.03	1.10	1.30x10^-4^	1.03	1.00	1.06	0.07	7.24x10^-3^
Essential hypertension	1.06	1.03	1.10	2.49x10^-4^	1.03	1.00	1.06	0.08	7.71x10^-3^
Hypertensive heart disease	1.15	1.08	1.23	2.07x10^-5^	1.05	0.98	1.13	0.16	1.19x10^-2^
Coronary atherosclerosis	1.06	1.02	1.10	1.40x10^-3^	1.02	0.98	1.07	0.31	0.14
Ischemic Heart Disease	1.05	1.01	1.09	8.19 x10^-3^	1.02	0.98	1.07	0.31	0.20
Loss of teeth or edentulism*	–	–	–	–	0.71	0.58	0.88	1.50x10^-3^	0.23
Obesity	1.08	1.03	1.13	2.72x10^-3^	1.07	1.03	1.11	1.06 x10^-3^	0.36
Type 2 diabetes	1.07	1.03	1.11	7.04x10^-4^	1.10	1.06	1.14	5.54 x10^-7^	0.37
Diabetes mellitus	1.06	1.03	1.10	6.78 x10^-4^	1.09	1.05	1.13	2.20 x10^-6^	0.52
Overweight, obesity and other hyperalimentation	1.08	1.03	1.13	9.74 x10^-4^	1.05	1.01	1.09	0.01	0.82

We used a logistic regression model to test the interaction of effect between PCOS_PRS_ and sex. The effect estimates for PCOS_PRS_ from the stratified models are shown for males and females. The interaction model was adjusted for the interaction term, PCOS_PRS_, age, sex, and genetic ancestry. *Males did not meet the minimum case requirement (n ≥ 100).

We also found that males were at an increased risk for hypertensive heart disease (p_interaction_ = 7.13x10^-6^) than females with the same BMI (**Table 2**). Together, these results indicate that sex is an important modifier of both PCOS genetic risk and BMI.

**Table 2:** Sex and BMI Significantly Modify Metabolic Associations Influenced by PCOS_PRS_.

	Males				Females				
Phenotype	OR	LCI	UCI	P	OR	LCI	UCI	p	Interaction P Value
Hypertensive heart disease	1.12	1.11	1.13	1.49 x10^-104^	1.10	1.09	1.11	3.09x10^-102^	7.13x10^-6^
Diabetes mellitus	1.11	1.10	1.11	6.63x10^-286^	1.10	1.09	1.10	< 1x10^-350^	0.01
Type 2 diabetes	1.12	1.11	1.12	< 1x10^-350^	1.11	1.10	1.11	< 1x10^-350^	0.02
Coronary atherosclerosis	1.05	1.04	1.05	5.27 x10^-53^	1.04	1.03	1.05	7.73 x10^-41^	0.11
Ischemic Heart Disease	1.04	1.04	1.05	3.71 x10^-48^	1.04	1.03	1.04	1.07 x10^-42^	0.21
Essential hypertension	1.08	1.07	1.09	3.91 x10^-188^	1.08	1.07	1.08	< 1x10^-350^	0.33
Hypertension	1.08	1.07	1.08	1.30 x10^-183^	1.08	1.07	1.08	< 1x10^-350^	0.40
Loss of teeth or edentulism*	–	–	–	–	1.00	0.98	1.03	0.87	0.59

We used a logistic regression model to test the interaction of effect between sex and body mass index (BMI). The effect estimates for BMI from the stratified models are shown for males and females. The interaction model was adjusted for the interaction term, sex, BMI, PCOS_PRS_, age, and genetic ancestry. *Males did not meet the minimum case requirement (n ≥ 100).

### Sensitivity analyses adjusting for PCOS case status, BMI, and BMI_residual_

In a separate sensitivity analysis (EUR-ancestry females only), we found that after adjusting for PCOS diagnosis, females with a high PCOS_PRS_ still demonstrated a significant positive phenome-wide association with T2D and diabetes mellitus (**S3 Fig**). Next, we adjusted for median BMI in all of the EUR PCOS_PRS_-PheWAS models. There were no surviving associations in the sex-combined model or stratified analyses (**S3 and S4 Figs**), suggesting BMI may mediate the pleiotropic effects observed via PCOS_PRS_. However, we observed almost no difference from the original results in the female stratified analysis when adjusting for BMI_residual_, the environmental component of BMI (**S5 Fig**). Indeed, T2D remained significantly associated with PCOS_PRS_ in females (OR = 1.10, 95% CI = 1.05–1.14, p = 5.45x10^-6^), as did diabetes mellitus (OR = 1.09, 95% CI = 1.04–1.13, p = 2.78x10^-5^) when adjusting for the BMI_residual_. These two associations also remained in the BMI_residual_ adjusted sex-combined results (**S6 Fig**). None of the associations passed Bonferroni correction in the male stratified analysis when adjusting for BMI_residual_ (**S5 Fig**).

### Genetic correlation results between PCOS and CMDs

BMI (r_g_ = 0.38, p = 7.81x10^-14^) and T2D (r_g_ = 0.29, p = 3.25x10^-9^) had the strongest genetic correlation with PCOS (**Table 3**). Heart failure (r_g_ = 0.26, p = 2.5x10^-3^) and pulse pressure (r_g_ = 0.13, p = 1.1x10^-2^) also had a modest genetic correlation with PCOS. CAD bordered a nominal significance threshold, and systolic and diastolic blood pressure were not significantly genetically correlated with PCOS.

**Table 3:** PCOS Shares Genetic Architecture with Cardiometabolic Traits.

Traits	Genetic Correlation	SE	Z	P
Diastolic Blood Pressure	-3.60%	0.07	-0.54	0.59
Systolic Blood Pressure	9.33%	0.05	1.79	0.07
Pulse Pressure	13.46%	0.05	2.54	0.01
Type 2 Diabetes	29.26%	0.05	5.92	3.25x10^-9^
Heart Failure	26.51%	0.09	3.02	0.003
Coronary Artery Disease	17.35%	0.09	1.95	0.05
Body Mass Index	38.40%	0.05	7.47	7.81x10^-14^

### CMD_PRS_ analysis of PCOS diagnosis

**Fig 4:**
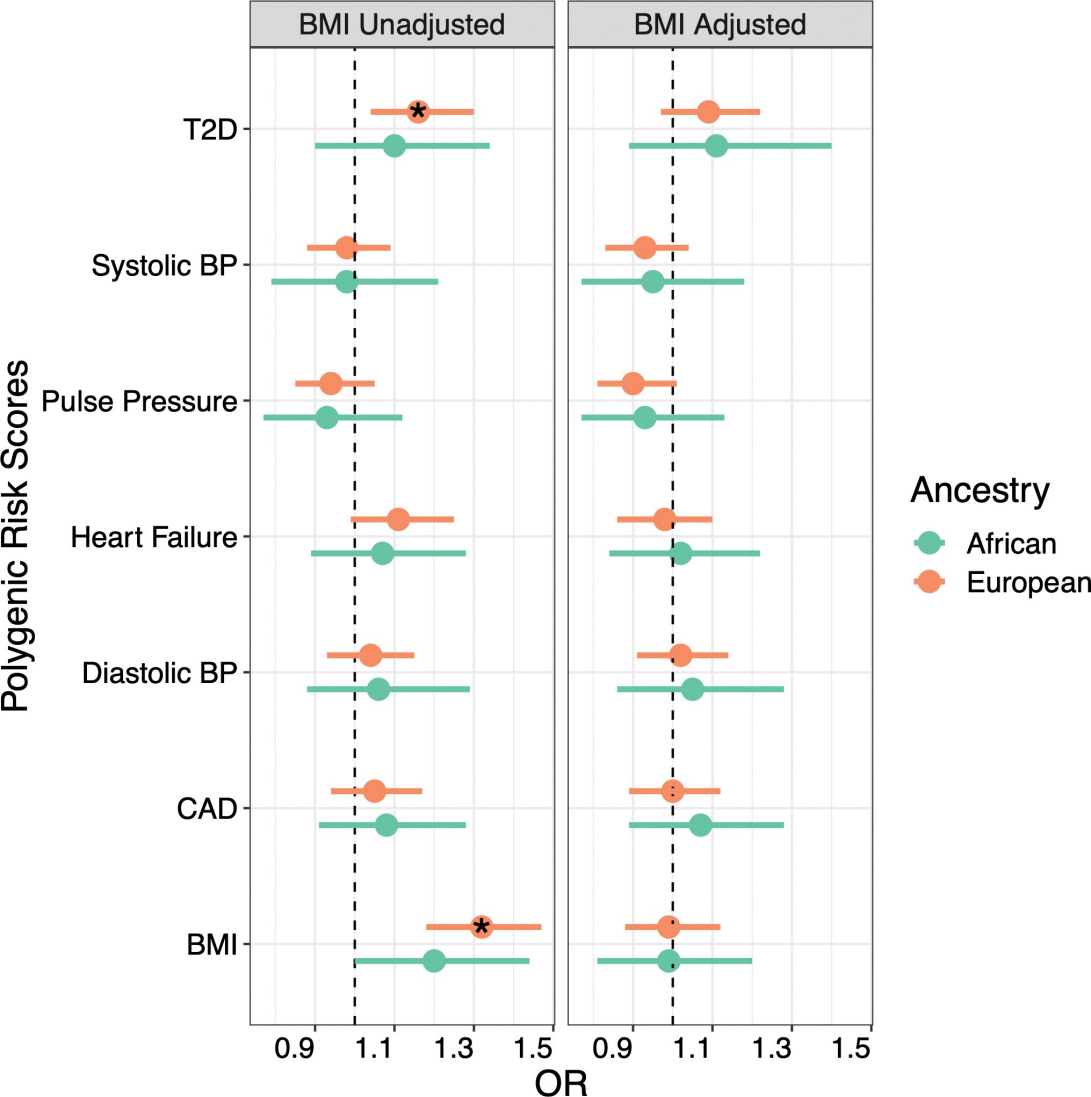
T2D_PRS_ and BMI_PRS_ are Associated with PCOS Case Status. The results from the logistic regression analysis between PCOS diagnosis and polygenic risk scores (PRS) for significant cardiometabolic traits from the PheWAS analysis are displayed in the forest plot. Results from the unadjusted and adjusted body mass index (BMI) models are shown. CAD = coronary artery disease, T2D = type 2 diabetes, BP = blood pressure. * indicates P < 0.05.

Outside of T2D and BMI, none of the PRS built for CAD, heart failure, or blood pressure traits were significantly associated with a PCOS diagnosis in females of either EUR or AFR-ancestry (**Fig 4**).

**Fig 5:**
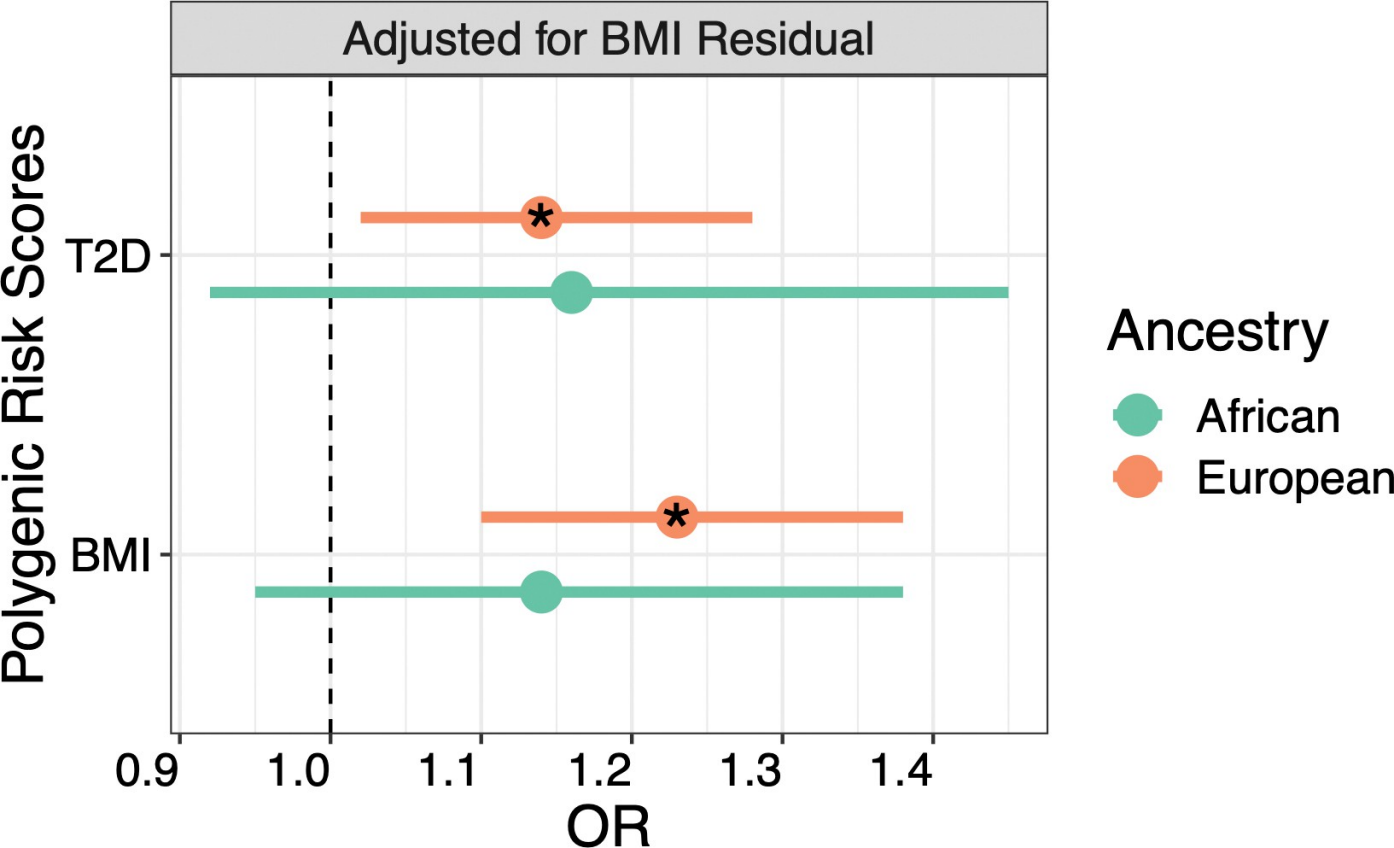
T2D_PRS_ is Associated with PCOS After Accounting for BMI_residual_. The logistic regression model between T2D_PRS_ and PCOS diagnosis was covaried for age, the top ten principal components for ancestry, and BMI_residual_. BMI_residual_ represents the environmental variance of BMI. * indicates P < 0.05.

BMI_PRS_ was positively associated with PCOS diagnosis in EUR females (OR = 1.32, 95% CI = 1.18–1.47, p = 6.13x10^-7^) and this finding was replicated in the AFR-ancestry population (OR = 1.20, 95% CI = 1.00–1.44, p = 0.05). However, the BMI_PRS_ was not significant after adjusting for clinically measured BMI. T2D_PRS_ also associated with PCOS diagnosis for EUR-ancestry females, again losing significance when conditioned on BMI (OR_unadjusted_ = 1.16, 95% CI = 1.04–1.30, p = 6.75x10^-3^; OR_adjusted_ = 1.09, 95% CI = 0.97–1.22, p = 0.16). To determine whether this reduction in effect size was due to the genetic correlation between T2D and BMI, we tested a model in which T2D_PRS_ was covaried for BMI_residual_ (**Fig 5**). This model indeed recovered the original association between T2D_PRS_ and PCOS diagnosis for the EUR population (OR = 1.14, 95% CI = 1.02–1.28, p = 0.02), suggesting that *genetically predicted* BMI, not BMI_residual_, mediates the association between T2D_PRS_ and PCOS diagnosis.

## Discussion

First, through a comprehensive analysis of PCOS genetic risk across multiple phenotypes, we identified sex differences in the cardiometabolic traits associated with PCOS genetic risk. Among these, the most notable difference was that males with high PCOS_**PRS**_ were at greater risk of cardiovascular conditions than females at the same level of genetic risk. Furthermore, only T2D_**PRS**_ and BMI_**PRS**_ were associated with PCOS diagnosis, indicating that many of the associations observed in the PCOS_**PRS**_ PheWAS were primarily driven by PCOS genetic risk and not the genetic effects of the identified comorbidities. Second, BMI also had strong effects on many of these associations with genetically regulated BMI being a primary driver of the risk between PCOS and T2D (**S7 Fig**).

There is growing interest in studying PCOS related effects in males whether through their relationship to first-degree family members with PCOS or by an equivalent phenotype [32–34]. Generally, males with mothers or sisters with PCOS tend to exhibit a poorer cardiometabolic profile which can be observed as early as infancy [33]. Previous studies suggest males who exhibit high genetic risk for PCOS are more likely to present with CMDs such as obesity, T2D, and diabetes mellitus, findings which were among the top associations for males in this study [12,35]. Our sex*PCOS_PRS_ interaction analysis further showed that males with a high genetic risk for PCOS had an increased risk for hypertension compared to females. Additionally, the sex*BMI interaction analysis demonstrated that males had a greater likelihood of hypertensive heart disease compared to females with the same BMI. We found that associations with CMD phenotypes in males were largely accounted for by genetically predicted BMI which can significantly increase the lifetime risk for CMD and mortality rates of high-risk individuals [36]. Genetic susceptibility for PCOS includes contributions from BMI and metabolic pathways. While the independent and sex-differential effects of BMI contribute to CMDs, these results also raise the hypothesis that sex hormones could play a role in the sex differential risk of CMDs conferred by the PCOS_PRS_. Together, these processes could be an additional catalyst for CMD events in males, making individuals already predisposed to adverse metabolic outcomes more vulnerable.

In an effort to determine whether genetic risk for phenotypes comorbid with PCOS could also increase risk for PCOS, we conducted separate multivariable logistic regressions with PRS for BMI, diastolic blood pressure, systolic blood pressure, pulse pressure, T2D, heart failure, and CAD on the PCOS diagnosis outcome and found no significant associations outside of T2D_PRS_ and BMI_PRS_. As with T2D, we found that genetically predicted BMI is primarily responsible for the association between T2D_PRS_ and PCOS, and for the reverse association between PCOS_PRS_ and T2D diagnosis. Given that females with PCOS are more likely to develop T2D at an earlier age, PCOS patients with a family history of T2D and obesity may have an even greater risk of morbid outcomes [37]. For example, this genetic susceptibility could explain the high prevalence of insulin resistance in PCOS patients, which can be as high as 70% regardless of BMI status [38]. Poor outcomes can also be further exacerbated by the genetic predisposition of BMI which already increases the risk of PCOS and T2D as shown by Mendelian Randomization studies [39,40]. Given that the environmental and genetically regulated variance of BMI can have differential effects on PCOS and resulting comorbidity risk, this study continues to underscore the importance of BMI in PCOS risk. Therefore, the repercussions of these effects should be investigated further, especially since the genetic and biological pathways could differ in lean PCOS patients who also experience a high rate of insulin resistance [41].

This study offers many strengths. Firstly, we showed that cardiometabolic associations vary with sex and that the metabolic outcomes related to PCOS genetic architecture can be further understood by studying both males and females. Secondly, we decomposed EHR measured BMI into genetically predicted (i.e., BMI_PRS_) and environmentally enriched (i.e., BMI_residual_) variance and evaluated their respective roles in mediating the cardiometabolic profiles associated with PCOS_PRS_. However, limitations include low power to detect any significant associations in our African ancestry sample. To date, there is no PCOS GWAS of African ancestry individuals, limiting all current similar studies to building PRS using European-based genetic variants, which do not perform as well in non-European populations [11,12]. Second, we only examined one environmental risk factor. Although many effects such as lifestyle and diet can be captured through BMI, it is not an exhaustive measurement nor does it accurately account for the full wellness of an individual [42,43]. Although other anthropometric features like hip-to-waist ratio (WHR) may be better indicators of health for some phenotypes [44], this information is not routinely collected in clinical settings or reported in EHRs. Furthermore, evidence does suggest that clinically ascertained BMI may be more informative for PCOS than WHR [39]. Finally, despite using the largest PCOS GWAS to date for this analysis, our PCOS_PRS_ still only explains a small portion of PCOS genetic variance. As these analyses expand, so too will our ability to detect the full genetic spectrum of PCOS and its subphenotypes.

PCOS is a multifaceted disorder with genetic architecture that is reflective of its heterogeneous outcomes. This polygenic structure captures a spectrum of metabolic comorbidities that is even more apparent when compared between sexes. Our findings show that males with high PCOS liability are indeed a high-risk group and those with a family history of PCOS should be closely monitored for hypertension and other CMDs. This is also true for females with PCOS and a family history of T2D, whose genetic risk could induce more severe comorbid outcomes. As such, management and screening strategies should be updated to reflect advances in PCOS etiology. This call to action is paramount and requires both widespread dissemination of risk factor information to relevant stakeholders and increases in PCOS research priorities and funding. This becomes even more crucial as PCOS comorbidities are often under-recognized in clinical settings and metabolic features are not included in many PCOS screening methods [45–48].

## Supporting information

S1 Fig Reproductive PCOS Symptoms are Best Predicted by the PCOS_PRS_ Constructed from Top GWAS Variants. A Bonferroni correction (P = 3.73x10^-5^) was applied to account for all tests in the phenome-wide association study of PCOS_PRS_ in European ancestry individuals. The red line represents the Bonferroni correction and the blue represents the false discovery rate of 0.05.
(TIFF)

S2 Fig PCOS_PRS_ is Underpowered in an African Ancestry Population. No associations passed Bonferroni correction (P = 8.14x10^-5^) for PCOS_PRS_ calculated in African ancestry individuals. The red line represents the Bonferroni correction and the blue represents the false discovery rate of 0.05.
(TIFF)

S3 Fig Pleiotropic Associations are Influenced by Phenotypic Features of PCOS. To determine the robustness of the observed significant associations with PCOS_PRS_, sensitivity analyses were performed in individuals of European ancestry. To identify what phenotypes were not the result of a PCOS diagnosis, (A) females were stratified and further adjusted for PCOS case status (Bonferroni correction P = 4.57x10^-5^). (B) PCOS_PRS_ was adjusted for BMI in the sex-combined dataset, but no associations passed the Bonferroni correction (P = 3.74x10^-5^) represented by the red line. The blue line represents a false discovery rate of 0.05.
(TIFF)

S4 Fig Associations Driven by PCOS_PRS_ Can Be Explained by BMI. Sex stratified analyses were adjusted for body mass index (BMI) in a sensitivity analysis. Results for European ancestry (A) females (P = 4.59x10^-5^) and (B) males (P = 5.07x10^-5^) are shown. The red line represents the Bonferroni correction and the blue represents the false discovery rate of 0.05.
(TIFF)

S5 Fig Accounting for BMI_residual_ Improves Top Associations. European ancestry sex stratified results were adjusted for BMI_residual_, age, and the top ten principal components for (A) females and (B) males. The Bonferroni correction was P = 4.59x10^-5^ for females and P = 5.07x10^-5^ for males. The red line represents the Bonferroni correction and the blue line represents the false discovery rate of 0.05.
(TIFF)

S6 Fig Type 2 Diabetes is Significant After Adjustment of BMI_residual_ in the Sex-Combined Model. The model was adjusted for BMI_residual_, age, and the top ten principal components in individuals of European ancestry. The red line represents the Bonferroni correction of P = 3.74x10^-5^ and the blue line represents the false discovery rate of 0.05.
(TIFF)

S7 Fig Project Summary. The diagram illustrates the main findings of the paper.
(TIFF)

S1 Supporting Information Results from the PheWAS analyses and the CMD_PRS_ regression analysis on PCOS diagnosis. (XLSX)
