# Supplementary figures and images for: Sex modifies the effect of genetic risk scores for polycystic ovary syndrome on metabolic phenotypes
Source: PLoS Genet. 2023 May 31;19(5):e1010764. doi: 10.1371/journal.pgen.1010764 (PMC10259776; doi:10.1371/journal.pgen.1010764)

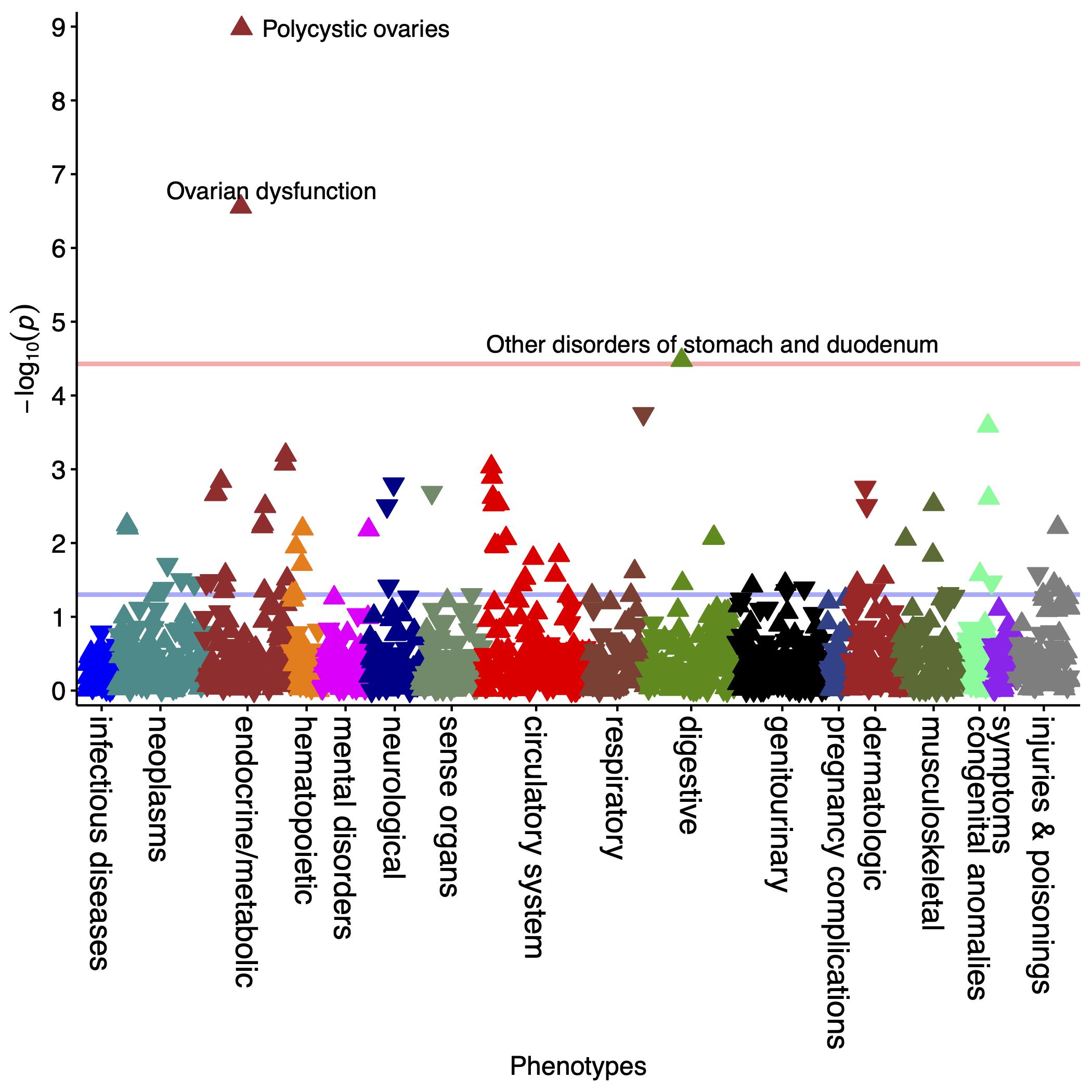

Supplement: S1 Fig — A Bonferroni correction (P = 3.73x10^-5^) was applied to account for all tests in the phenome-wide association study of PCOSPRS in European ancestry individuals. The red line represents the Bonferroni correction and the blue represents the false discovery rate of 0.05. (TIFF) [file pgen.1010764.s001.tiff]

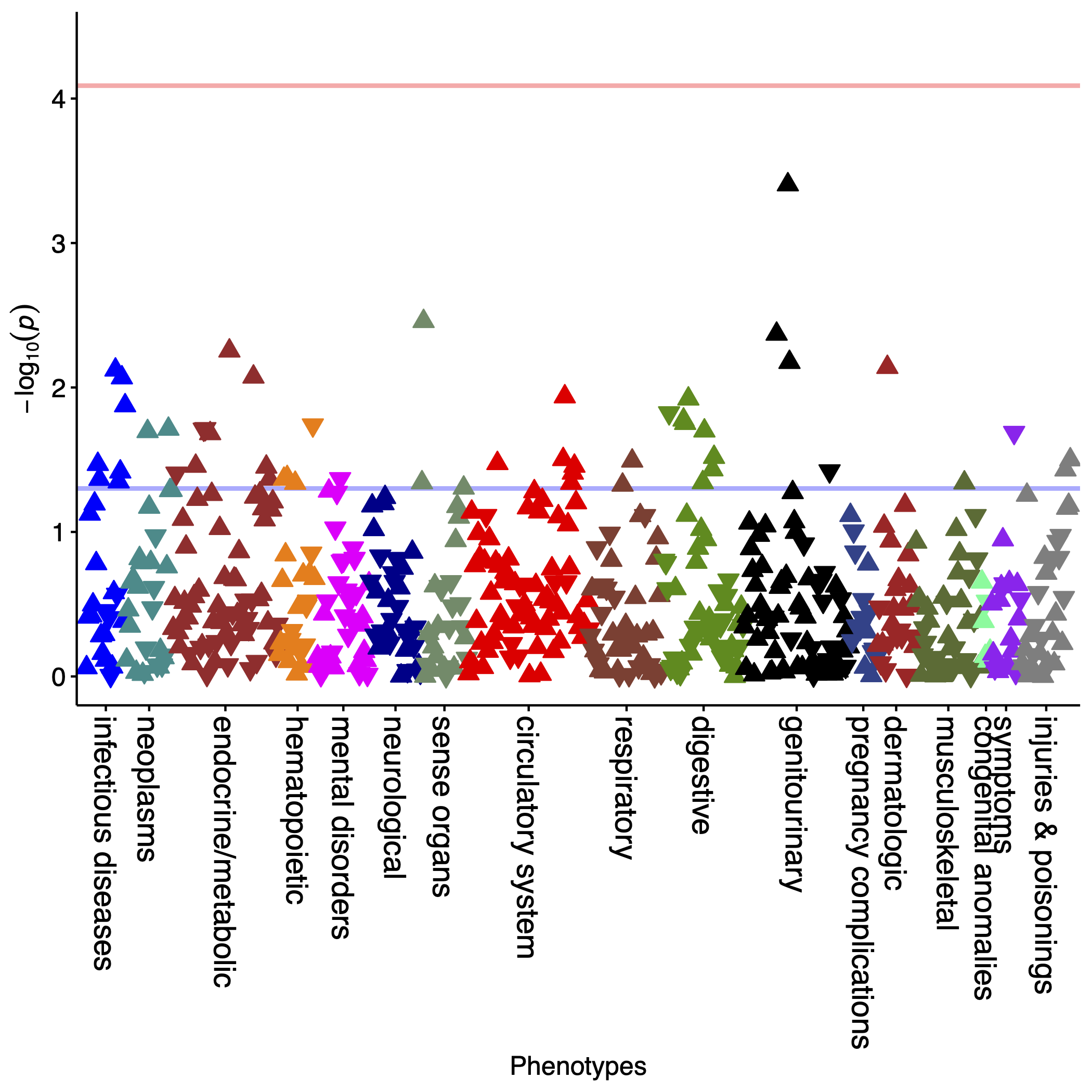

Supplement: S2 Fig — No associations passed Bonferroni correction (P = 8.14x10^-5^) for PCOSPRS calculated in African ancestry individuals. The red line represents the Bonferroni correction and the blue represents the false discovery rate of 0.05. (TIFF) [file pgen.1010764.s002.tiff]

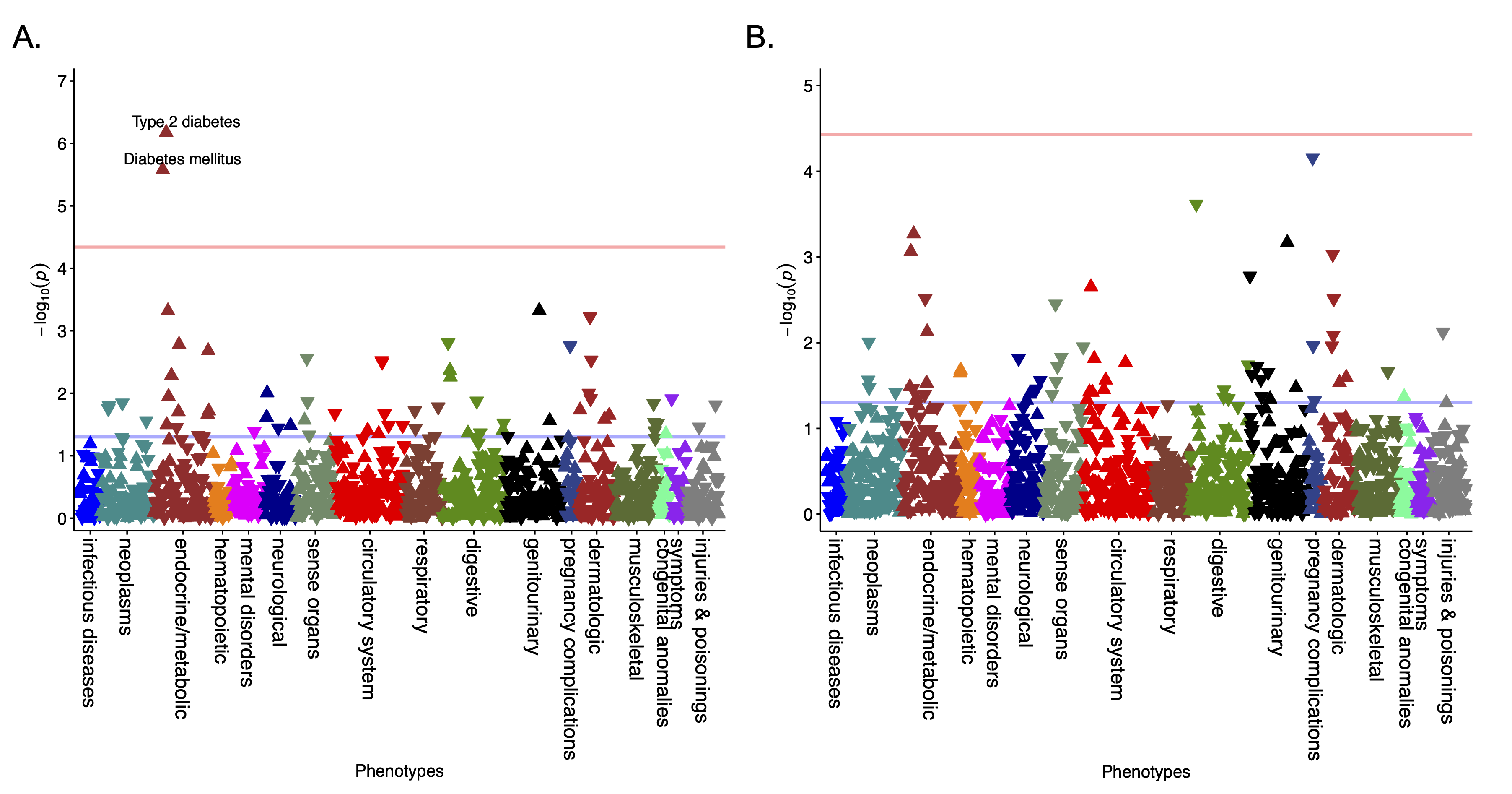

Supplement: S3 Fig — To determine the robustness of the observed significant associations with PCOSPRS, sensitivity analyses were performed in individuals of European ancestry. To identify what phenotypes were not the result of a PCOS diagnosis, (A) females were stratified and further adjusted for PCOS case status (Bonferroni correction P = 4.57x10^-5^). (B) PCOSPRS was adjusted for BMI in the sex-combined dataset, but no associations passed the Bonferroni correction (P = 3.74x10^-5^) represented by the red line. The blue line represents a false discovery rate of 0.05. (TIFF) [file pgen.1010764.s003.tiff]

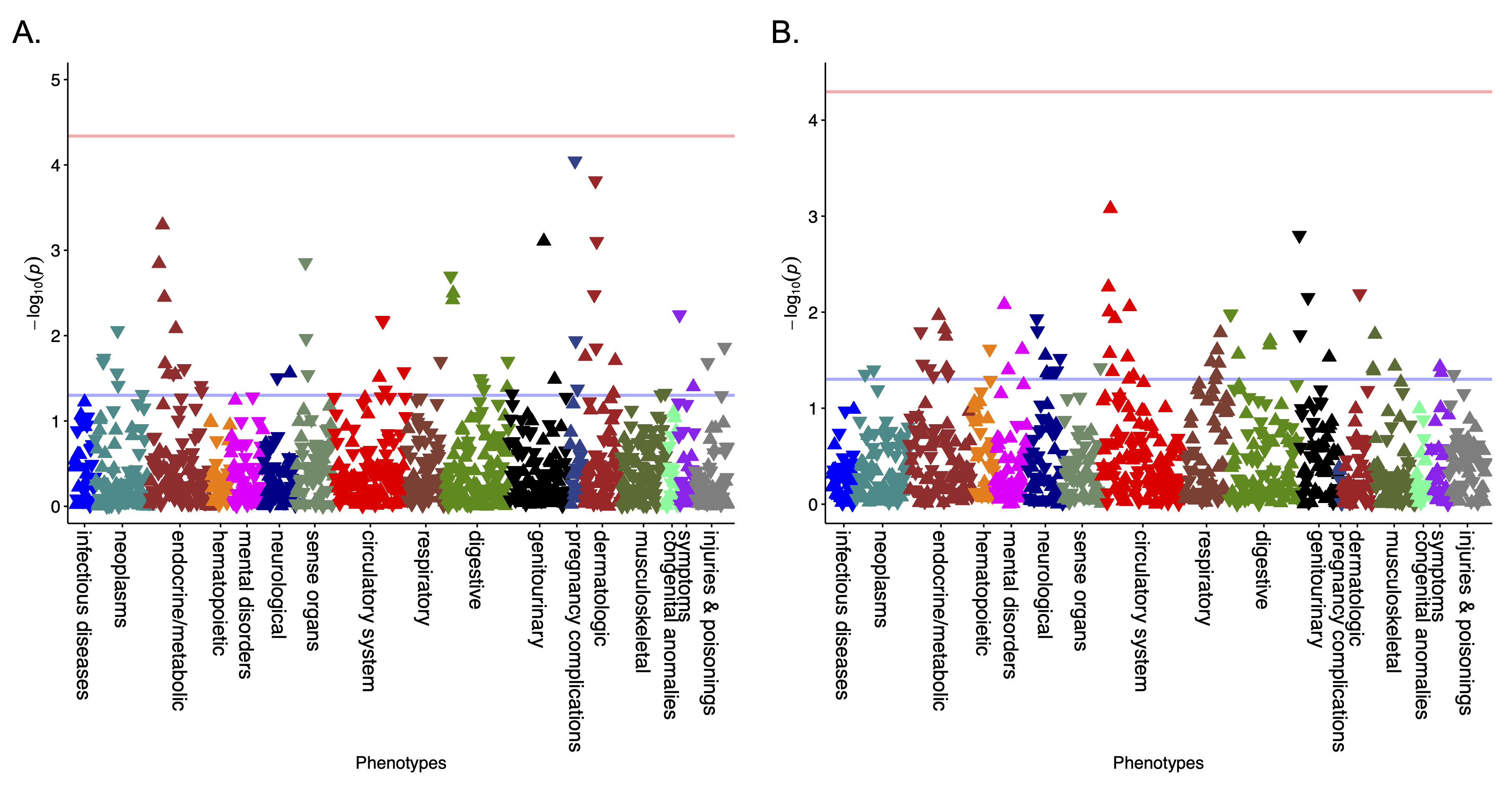

Supplement: S4 Fig — Sex stratified analyses were adjusted for body mass index (BMI) in a sensitivity analysis. Results for European ancestry (A) females (P = 4.59x10^-5^) and (B) males (P = 5.07x10^-5^) are shown. The red line represents the Bonferroni correction and the blue represents the false discovery rate of 0.05. (TIFF) [file pgen.1010764.s004.tiff]

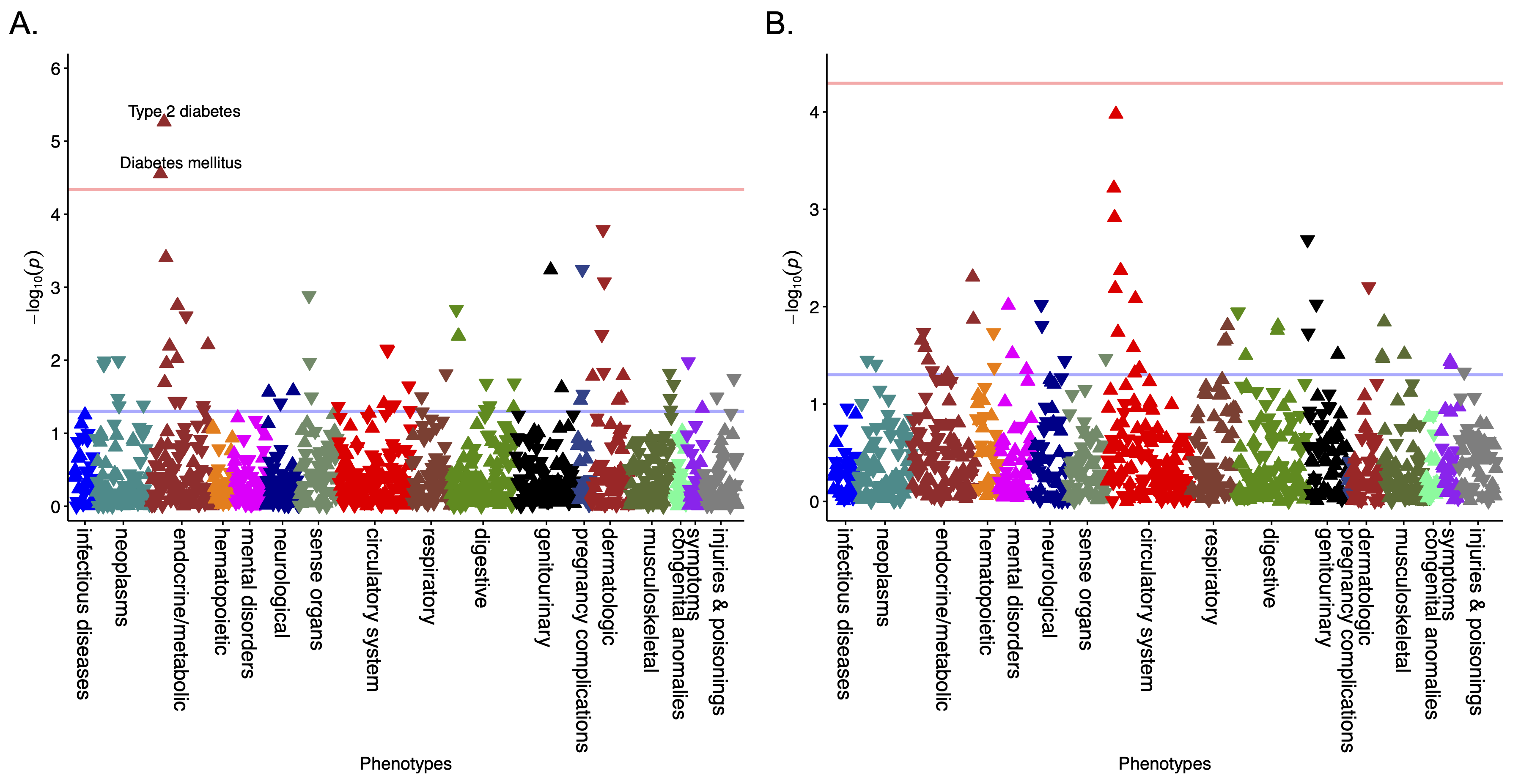

Supplement: S5 Fig — European ancestry sex stratified results were adjusted for BMIresidual, age, and the top ten principal components for (A) females and (B) males. The Bonferroni correction was P = 4.59x10^-5^ for females and P = 5.07x10^-5^ for males. The red line represents the Bonferroni correction and the blue line represents the false discovery rate of 0.05. (TIFF) [file pgen.1010764.s005.tiff]

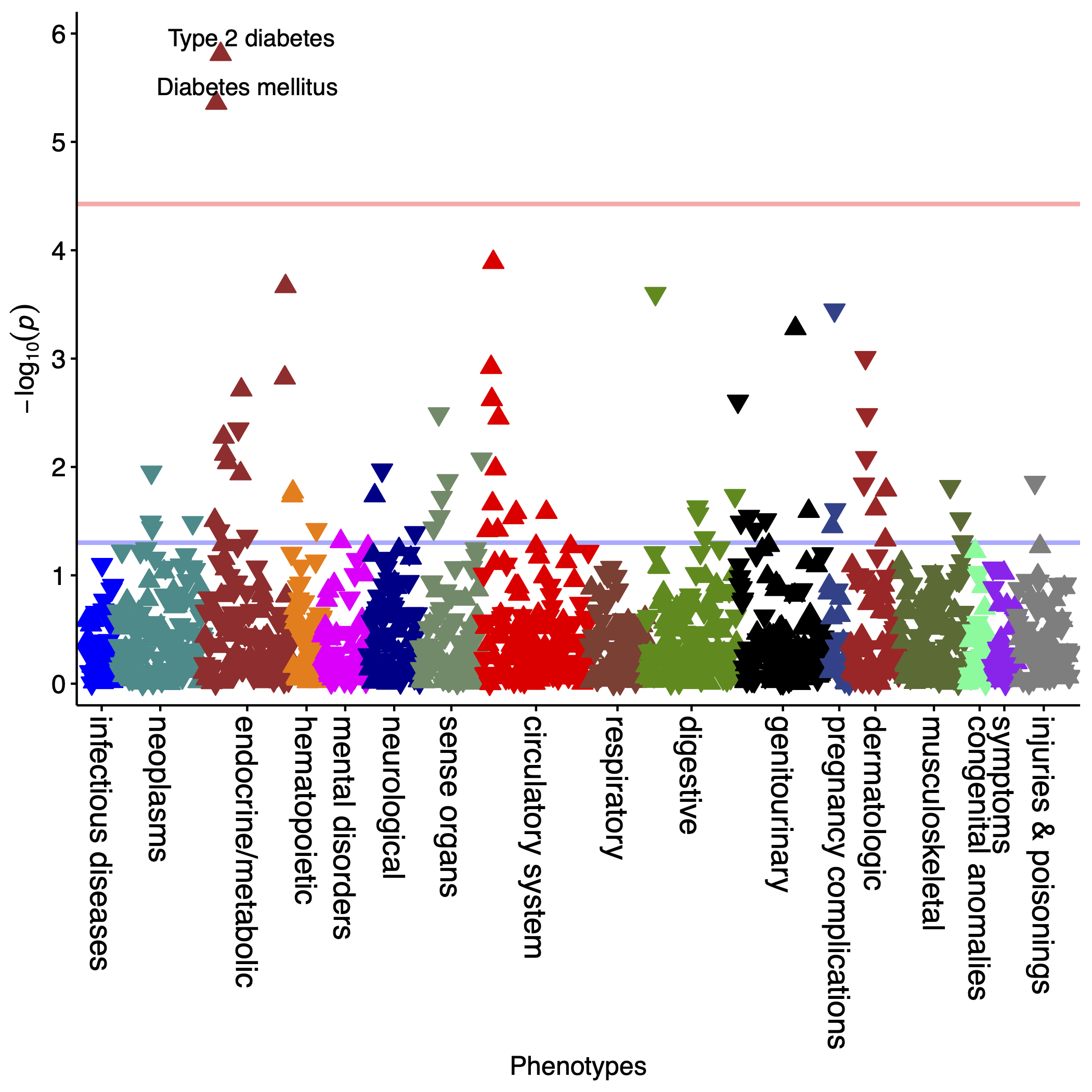

Supplement: S6 Fig — The model was adjusted for BMIresidual, age, and the top ten principal components in individuals of European ancestry. The red line represents the Bonferroni correction of P = 3.74x10^-5^ and the blue line represents the false discovery rate of 0.05. (TIFF) [file pgen.1010764.s006.tiff]

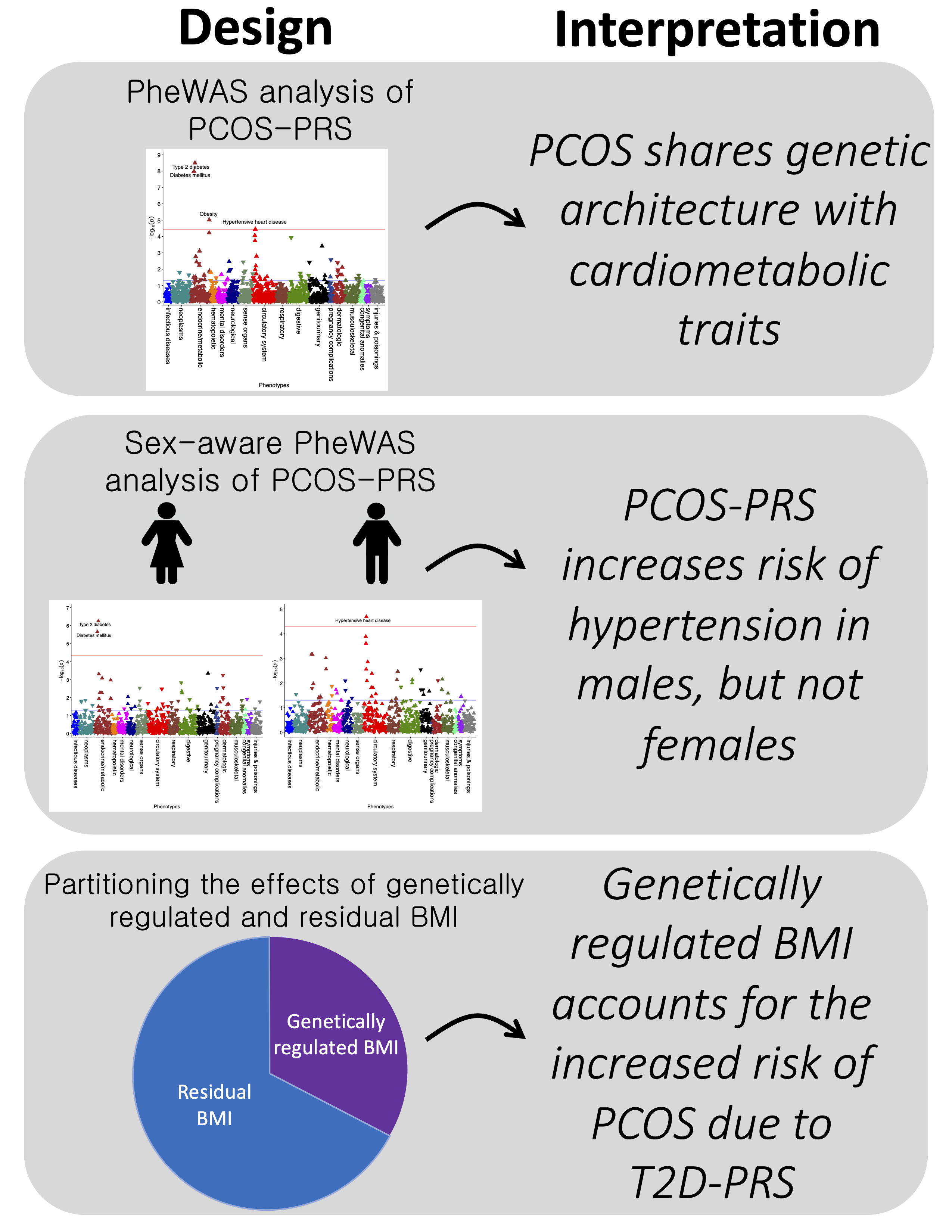

Supplement: S7 Fig — The diagram illustrates the main findings of the paper. (TIFF) [file pgen.1010764.s007.tiff]
